# Disgraceful "Ladies"

**Published:** 1957-01

**Authors:** Winifred Tanner


					Correspondence
DISGRACEFUL "LADIES"
"ear Sir,
. May I ask if something .an be done aboutZ?t^^ seaM'
J have recently been on a motoring holiday and w ^ ^ ^
?wns the public conveniences were_no' .(c %vith ,hose great anti-dise
The condition of them is hard to rec or.pnt to improve
esrl?f the campaign launched against disea . little money could P . . which are
They are in a disgraceful state and one does?eelthat ^ ^ country-amenities wfti
?hem instead of providing so many ameni ics pvnmole of what
??* available to us when we are abroad. Exeter which is a glowing
We have a public convenience in the cen
should be done?it is always spotless.
, 1 write this in the interest of public hea . sincerely,
Exeter.
Winifred Tanner

				

